# Foot Dimension Assessment: Reliability and Agreement of Manual, Pedobarographic, and Handheld 3D Scanning Methods

**DOI:** 10.3390/jcm15010100

**Published:** 2025-12-23

**Authors:** Lennart Schleese, Thomas Mittlmeier, Dagmar-C. Fischer, Paul Abshagen, Jonas Opfermann, Patrick Gahr, Martin Behrens, Sven Bruhn, Matthias Weippert

**Affiliations:** 1 Department of Traumatology, Hand- and Reconstructive Surgery, Rostock University Medical Center, 18057 Rostock, Germany; 2 Department of Orthopaedics, Rostock University Medical Center, 18057 Rostock, Germany; 3 University of Applied Sciences Sports and Management, 14471 Potsdam, Germany; 4 Institute of Sport Science, University of Rostock, 18057 Rostock, Germanymatthias.weippert@uni-rostock.de (M.W.)

**Keywords:** 3D scanner, pedobarography, foot dimensions, footprint angles, intra-rater reliability

## Abstract

**Background**: Accurate assessment of foot morphology is essential in sports medicine, orthopaedics, and footwear design. Manual examination remains common but may lack accuracy and reproducibility. Alternative techniques, such as pedobarography and handheld 3D scanning, may offer more objective and reliable data, given that their reliability and agreement with established methods are confirmed. **Methods**: Twenty-six healthy adults (age 25.8 ± 4.7 years; BMI 24.1 ± 2.0 kg/m^2^) were investigated. Foot dimensions were assessed via manual examination, pedobarography, and handheld 3D scanning, each performed in random order by two independent investigators on two separate occasions. Relative and absolute intra-rater reliability were analysed using intraclass correlation coefficients (ICC), the change in the mean of repeated measurements (bias), limits of agreement (LoA), and the typical error (TE). Inter-method agreement was evaluated using Lin’s concordance correlation coefficients (CCC), mean bias, and LoA to assess interchangeability as well as systematic bias. **Results**: Good-to-excellent relative and absolute intra-rater reliability was found for the distance-related foot dimensions across all methods, except for heel width assessed via pedobarography (small bias but wide LoA and high TE). Relative and absolute reliability of the angular parameters assessed via pedobarography and 3D scanning ranged from poor to excellent. Inter-method agreement between manual examination, pedobarography, and 3D scanning appeared low when considering all three agreement indices (i.e., CCC, mean bias, and LoA). The largest discrepancies were observed for heel width and arch-related measures. **Conclusions**: All three methods seem reliable for assessing distance-related foot dimensions. However, limited agreement among the three methodological approaches indicates that they cannot be used interchangeably without standardisation.

## 1. Background

The human foot is indispensable for standing and locomotion, yet it often only becomes the focus of clinical attention when pain, morphological deviations, or impaired gait are present [1]. Its internal and external structures are highly complex, and accurate assessment of its outer dimensions remains challenging due to the foot’s irregular geometry and dynamic adaptation under load. Excessive body weight, repetitive exposure to ground reaction forces, or poorly fitting footwear may alter foot structures and exacerbate functional problems such as hallux valgus or arch deformities [1,2,3].

Many morphological abnormalities are clinically evident and can be diagnosed by simple visual inspection and manual examination. Quantitative assessment typically involves measuring geometric parameters such as foot length, forefoot and heel width, arch height, or angular measures using rulers or goniometers. However, these classical anthropometric methods strongly depend on the examiner’s experience [4]. Quantitative foot assessment is further complicated by irregular surface geometry, curved measurement paths, and load-dependent variation in plantar contact with the ground, all of which may impair measurement precision [4].

Pedobarographic platforms and devices have been widely applied to analyse postural stability, plantar loading patterns, and foot-related musculoskeletal conditions. Moreover, a systematic review has shown that pedobarography is frequently used to quantify balance parameters and plantar pressure distribution in healthy individuals [5,6]. Clinical applications include evaluating weight distribution and functional recovery following lower-limb injuries, such as calcaneal fractures treated with the Ilizarov method, where pedobarography helps to identify asymmetries and compensatory mechanisms [7].

While manual examination is widely used in clinical settings, more advanced approaches such as pedobarography and 3D scanning have been introduced to improve measurement accuracy and reproducibility. Pedobarography has also proven valuable for monitoring diabetic foot ulcers and characterising foot morphology [8,9]. By recording pressure distribution at the plantar surface, it provides digital 2D images from which footprint dimensions under weight-bearing, angular parameters, and arch-related indices can be derived [3].

In recent years, optical 3D scanning has gained attention as a method for generating accurate models of irregularly shaped structures. Such systems provide detailed surface geometry and volume data and are increasingly being used in footwear design and orthotics manufacturing [10,11,12,13]. Stationary 3D scanners have been shown to have a good relative and absolute intra- and inter-rater reliability for foot measurements [3,12,13], but they are bulky, static, and restricted to specialised laboratory settings. Handheld 3D scanners provide greater flexibility but have mostly been used to assess body-part volume under non-weight-bearing conditions.

To optimize the measurement of 3D scanning data, we developed a custom-made transparent, non-reflective polymethylmethacrylat platform that enables weight-bearing scanning with a handheld 3D device. The platform provides a stable, deformation-resistant interface for structured-light scanning, allowing complete foot geometry to be captured under physiological loading conditions and enabling the extraction of footprint geometry, angular parameters, and arch indices.

The purpose of this study was to evaluate the relative and absolute intra-rater reliability of handheld 3D scanning for weight-bearing foot measurements and to determine the level of agreement between 3D scanning and established assessment methods, namely, manual examination and pedobarography. We hypothesised that handheld 3D scanning would demonstrate high reliability for linear foot dimensions and acceptable agreement with the reference methods.

## 2. Methods

### 2.1. Participants and Study Procedure

The study was approved by the local ethics committee (registration no. A 2020-0176) and was conducted in accordance with the Declaration of Helsinki. Written informed consent was obtained from all participants prior to enrolment.

Healthy male sports students, aged 18–35 years and regularly engaged in recreational physical activity, were recruited and assessed in the investigation rooms of Rostock University Medical Center. Female participants and individuals with known neurological or neuromuscular degenerative disorders were excluded. Anthropometric data were collected at the baseline visit. Foot dimensions were assessed under weight-bearing conditions using each method twice for both feet, with a one-week interval between sessions. All pedographic measurements were conducted by one examiner (JO), while all 3D scans and manual examinations were performed by the second examiner (PA). Pooled data of the left and right feet from both sessions were used to calculate intra-rater test–retest reliability, whereas pooled values of the left and the right feet from the second session were used to determine agreement between the two measurement methods.

### 2.2. Study Examinations

#### 2.2.1. Manual Assessment of Foot Geometry

**Figure 1:**
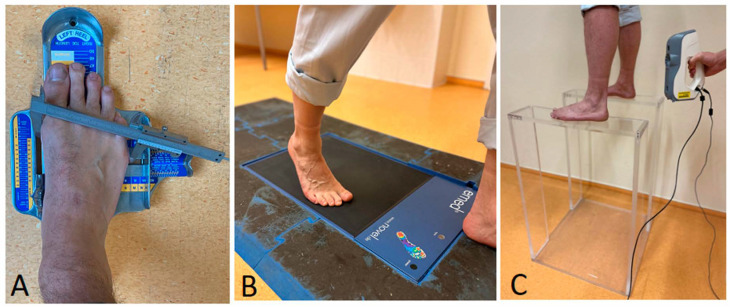
(**A**)—Manual examination of foot length (WMS1 foot measurement system, Offenbach, Germany) and forefoot width (caliper), (**B**)—Pedobarography with emed^®^ q pressure platform (novel gmbh, Munich, Germany), (**C**)—Acryl glass platform for scanning the footprints with a handheld 3D scanner (Artec Eva^©^, Luxembourg).

Foot length (the maximum distance between the heel and toe), forefoot width (the distance between the heads of the first and fifth metatarsals), and heel width (distance between the most medial and most lateral points of the calcaneal tuberosity) were measured using a standardised foot measurement system (WMS1, Offenbach, Germany) and a caliper (Mannesmann, Düsseldorf, Germany), while participants stood barefoot (see Figure 1A) [14]. Data were recorded in a spreadsheet separately for the right and left feet, as well as for the averaged values.

#### 2.2.2. Dynamic Pedobarography

Dynamic pedobarography was performed using the emed^®^ Q pressure platform (novel GmbH, Munich, Germany), in combination with proprietary software (emed^®^ medical professional 13.3.42d, novel GmbH, Munich, Germany), for data acquisition and automated calculation of foot dimensions. The emed^®^ system has been validated in previous research and is considered a reliable and accurate tool for assessing plantar pressure distribution and dynamic foot parameters [15]. The system consists of a platform with a surface area of 700 mm × 403 mm containing a sensor mat with calibrated capacitive pressure sensors (4 sensors/cm^2^; sampling rate 50 Hz).

**Figure 2:**
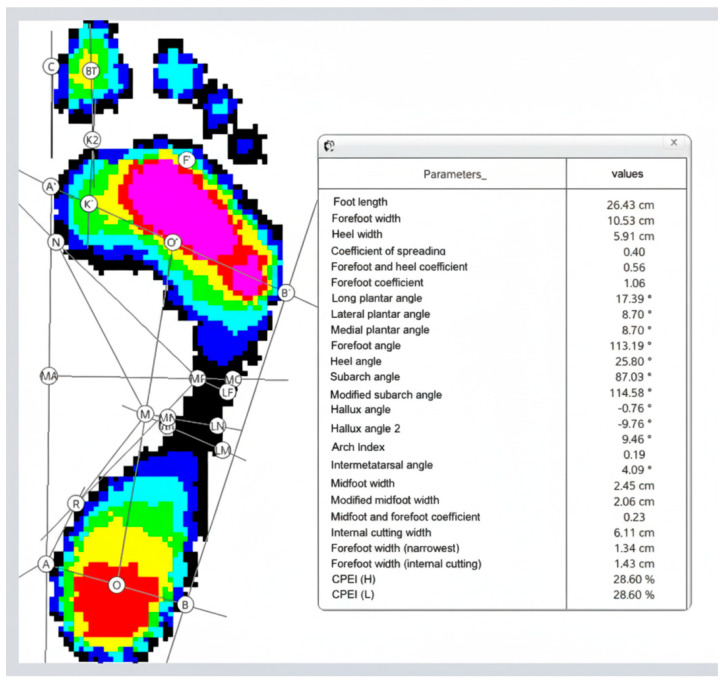
Model of the lower foot and the corresponding maximum pressure image obtained through dynamic pedobarography. The physiological footprint is depicted as isobars (red and violet indicating high pressure; blue indicating low pressure), with internal lines representing geometric parameters derived from the emed^®^ medical professional (novel GmbH, Munich, Germany).

Foot pressure was recorded during barefoot walking at a self-selected comfortable velocity. For each participant, data from four to five steps per foot were collected (see Figure 1B) and used to generate digital footprints. Based on these images, the software calculated the following parameters: foot length, forefoot width, heel width, subarch angle, and intermetatarsal angle. Body height and mass were also incorporated into the calculations (Figure 2).

#### 2.2.3. Three-Dimensional Optical Scanning of the Foot

A handheld 3D optical scanner (Artec Eva^®^, Artec3D, Luxembourg; maximum 3D resolution 0.2 mm, point accuracy 0.1 mm, working distance 0.4–1.0 m, reconstruction rate 4 fps) was used in combination with proprietary software (Artec Studio Professional 16, Artec3D, Luxembourg). Participants stood upright on a custom-designed polymethylmethacrylate platform (10 mm thickness, 0.9 m height) during the scanning procedure. Data from the dorsal and plantar aspects of the feet were merged into a 3D model (Figure 1C).

**Figure 3:**
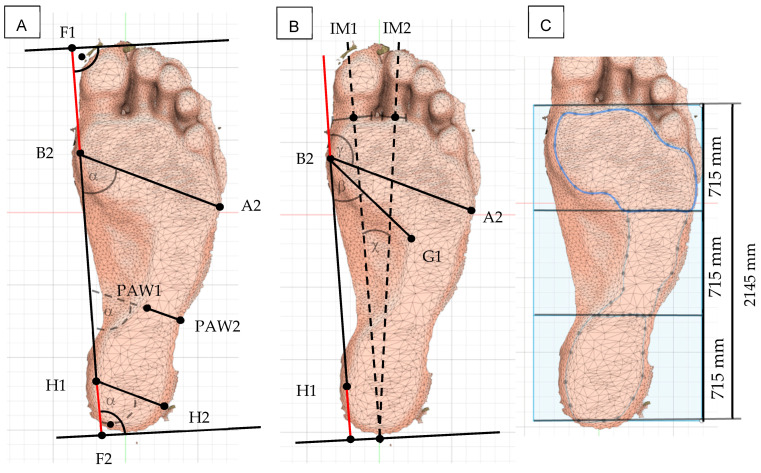
Model of the lower foot obtained using 3D scanning. (**A**) Distances and widths derived from the 3D model according to Mauch et al. [12]: foot length (F1–F2), forefoot width (B2–A2), plantar arch width (PAW1–PAW2), and heel width (H1–H2). (**B**) Angles: forefoot angle (γ = B2–A2 and extended B2–H1), subarch angle (β = B2–A2 and B2–H1), and intermetatarsal angle (χ = IM1–IM2). (**C**) The toeless forefoot area (a + b + c) and the areas of the forefoot (a), midfoot (b), and heel (c) are indicated [17].

Foot length, forefoot width, heel width, subarch angle, and intermetatarsal angle were calculated from the plantar model according to Mauch et al. [10,16], using Autodesk^®^ Fusion 360™ (Autodesk, Inc., Farnborough, UK) (Figure 3). Additionally, the area creator tool in the software was used to determine the toe-excluded footprint area and the area of its middle third to calculate the Arch Index (AI) (Figure 3) [17].

### 2.3. Data Handling and Statistical Analysis

Data processing was performed using the proprietary software provided with the pedobarography system (emed^®^ medical professional 13.3.42d, novel GmbH, Munich) and the 3D scanner (Artec Studio Professional 16, Artec3D, Luxembourg), in combination with Autodesk^®^ Fusion 360 (Autodesk, Inc., Farnborough, UK). Additional analyses were carried out using Microsoft Excel (version 2009, Microsoft Corporation, Redmond, WA, USA), SigmaPlot^®^ (version 13, Systat Software, Inc., San Jose, CA, USA) and SPSS Statistics (version 27, IBM Corporation, Chicago, IL, USA).

Data distribution was assessed for normality using the Shapiro–Wilk test. Continuous variables are reported as means ± standard deviations (SD) or as medians and ranges, if appropriate. Relative intra-rater reliability was evaluated using the 95% confidence intervals (CI) of the intraclass correlation coefficient (ICC) values. A CI greater than 0.90 was considered to indicate excellent reliability; values between 0.75 and 0.90 indicated good reliability, and values between 0.50 and 0.75 indicated moderate reliability [18,19,20]. Absolute intra-rater reliability was assessed with the change in the mean of repeated measurements (bias), limits of agreement, and the typical error of the test–retest measurements [18,19,20].

Inter-method agreement was evaluated with Lin’s Concordance Correlation Coefficient (CCC), Bland–Altman analysis to assess interchangeability, and systematic bias [20,21,22,23,24]. For the CCC calculation an Excel spreadsheet downloaded from https://real-statistics.com/reliability/interrater-reliability/lins-concordance-correlation-coefficient/ (accessed on 30 November 2025) was used. CCC values ≤ 0.20 are interpreted as poor; values ≥ 0.80 represent excellent agreement, and values in between indicate moderate agreement [24].

## 3. Results

Twenty-eight healthy, physically active male students consented to participate. Measurements were incomplete for two participants, so the final analysis included 26 participants (age: 23.5 ± 4.7 years; height: 1.85 ± 0.10 m; body mass: 82.3 ± 8.9 kg; BMI: 24.1 ± 2.0 kg/m^2^).

For each participant and method, data from the left and the right feet were pooled (i.e., N = 52) and used to assess relative and absolute intra-rater test–retest reliability (Table 1). To investigate agreement between methods, the pooled values from the second examination were analysed.

### 3.1. Relative and Absolute Intra-Rater Reliability

Statistics for relative and absolute intra-rater reliability of the manual examination, pedobarography, and 3D scan data are displayed in Table 2.

Manual examination achieved excellent relative reliability for the linear foot dimensions (ICC lower 95% CI: 1.0). Absolute reliability revealed a significant but small bias, narrow limits of agreement, and a low typical error for foot length and even smaller non-significant biases, narrower limits of agreement, and lower typical error for forefoot as well as heel width.

Pedobarography showed moderate to excellent relative reliability for linear parameters (ICC lower 95% CI ranged from 0.6–1.0) and poor to excellent reliability for arch- and angle-related measures (ICC lower 95% CI ranged from −0.2–1.0).

Absolute reliability revealed no bias, narrow limits of agreement, and a low typical error for foot length and forefoot width. However, despite an acceptable bias < 1 mm for heel width, limits of agreement appeared wide and typical error substantial, respectively. Angular parameters showed no significant bias but wide limits of agreement, which question the absolute reliability of pedobarographic measurements for these dimensions. However, absolute reliability appeared good for the arch index.

Handheld 3D scanning demonstrated good-to-excellent relative test–retest reliability for linear foot dimensions (ICC lower 95% CI ranged from 0.7–1.0). Angular parameters, except the subarch angle (ICC lower 95% CI: 0.6), and the arch index exhibited low relative reliability (ICC lower 95% CI ranged from 0.5 for the arch index to 0.1 for the forefoot angle). Absolute reliability revealed no bias, narrow limits of agreement, and a small typical error for foot length. In contrast, a significant, but small bias was found for forefoot width and a non-significant bias but large limits of agreement and a substantial typical error for the test–retest measurements of heel width using 3D scanning. The subarch and forefoot angles showed no significant bias, but wide limits of agreement and intermetatarsal angle test–retest measurements exhibited a substantial and systematic bias. These findings question the absolute reliability of 3D measurements for these dimensions. In contrast, absolute reliability appeared good for the arch index because there was no relevant and significant bias, narrow limits of agreement, and a small typical error for the test–retest assessment.

### 3.2. Agreement Between the Manual Examination, Pedobarography, and 3D Scan Data

**Figure 4:**
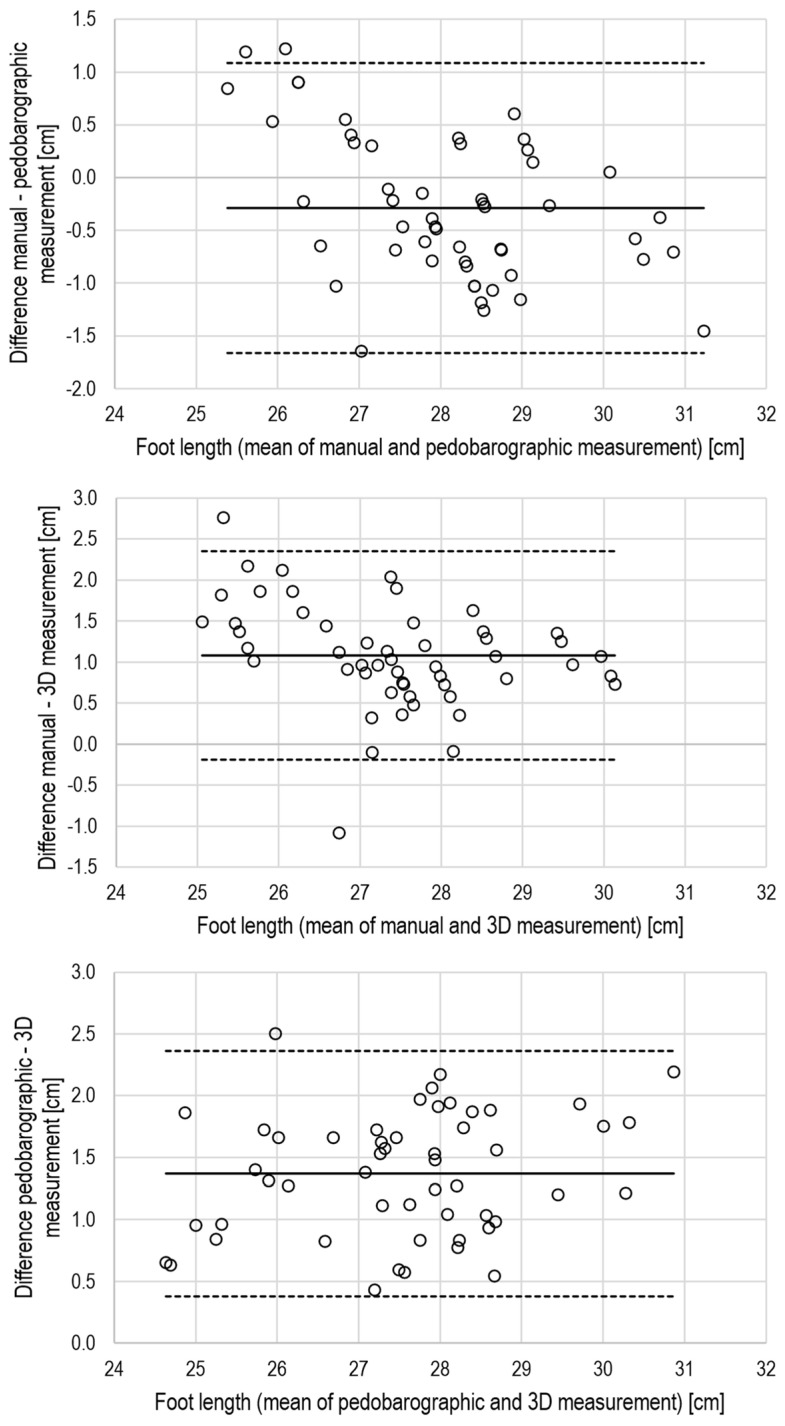
Bland–Altman plots for the evaluation of inter-method agreement for foot length, solid lines: mean difference, dashed lines: limits of agreement (mean ± 2 × standard deviation), upper panel: manual vs. pedobarography, middle panel: manual vs. 3D scanning, lower panel: pedobarography vs. 3D scanning.

**Figure 5:**
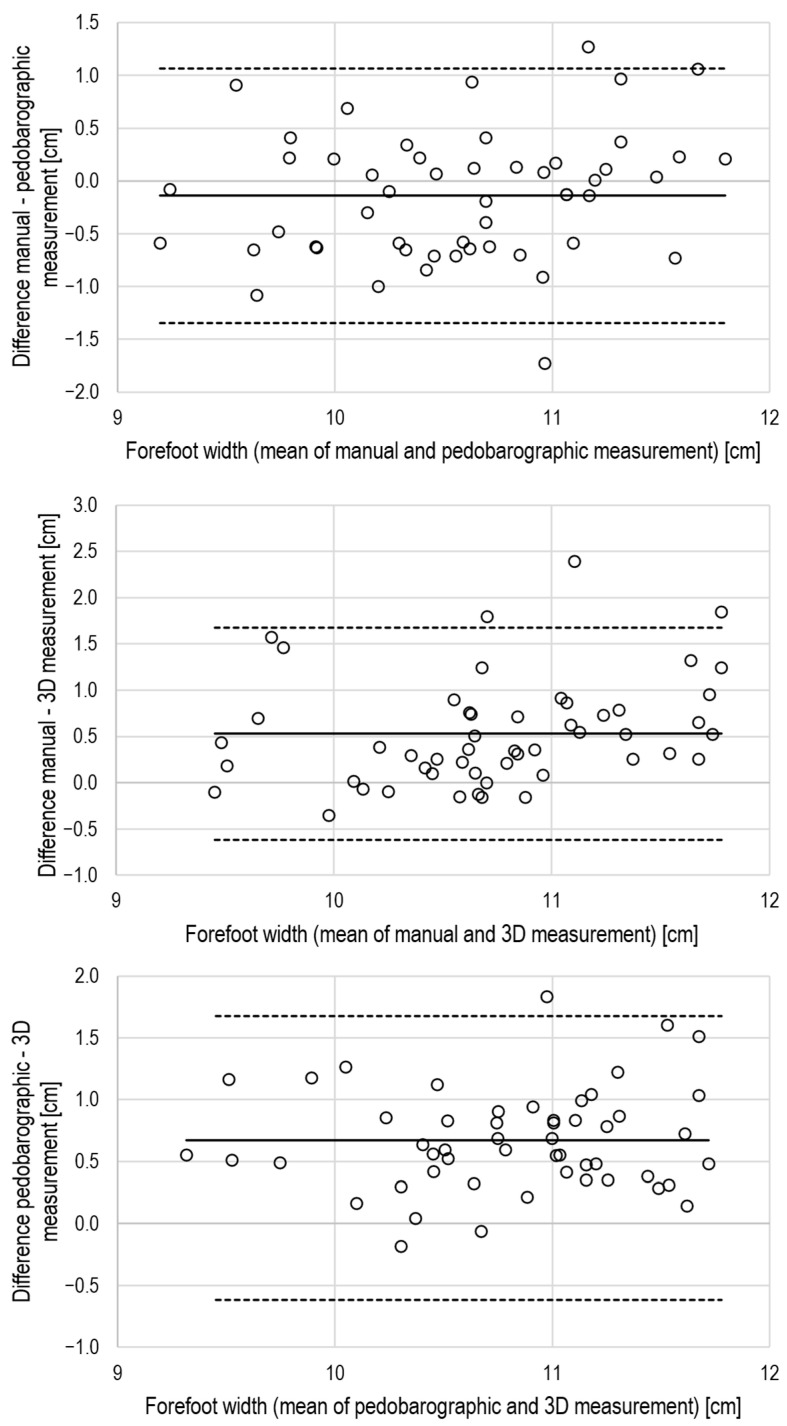
Bland–Altman plots for the evaluation of inter-method agreement for forefoot width, solid lines: mean difference, dashed lines: limits of agreement (mean ± 2 × standard deviation), upper panel: manual vs. pedobarography, middle panel: manual vs. 3D scanning, lower panel: pedobarography vs. 3D scanning.

**Figure 6:**
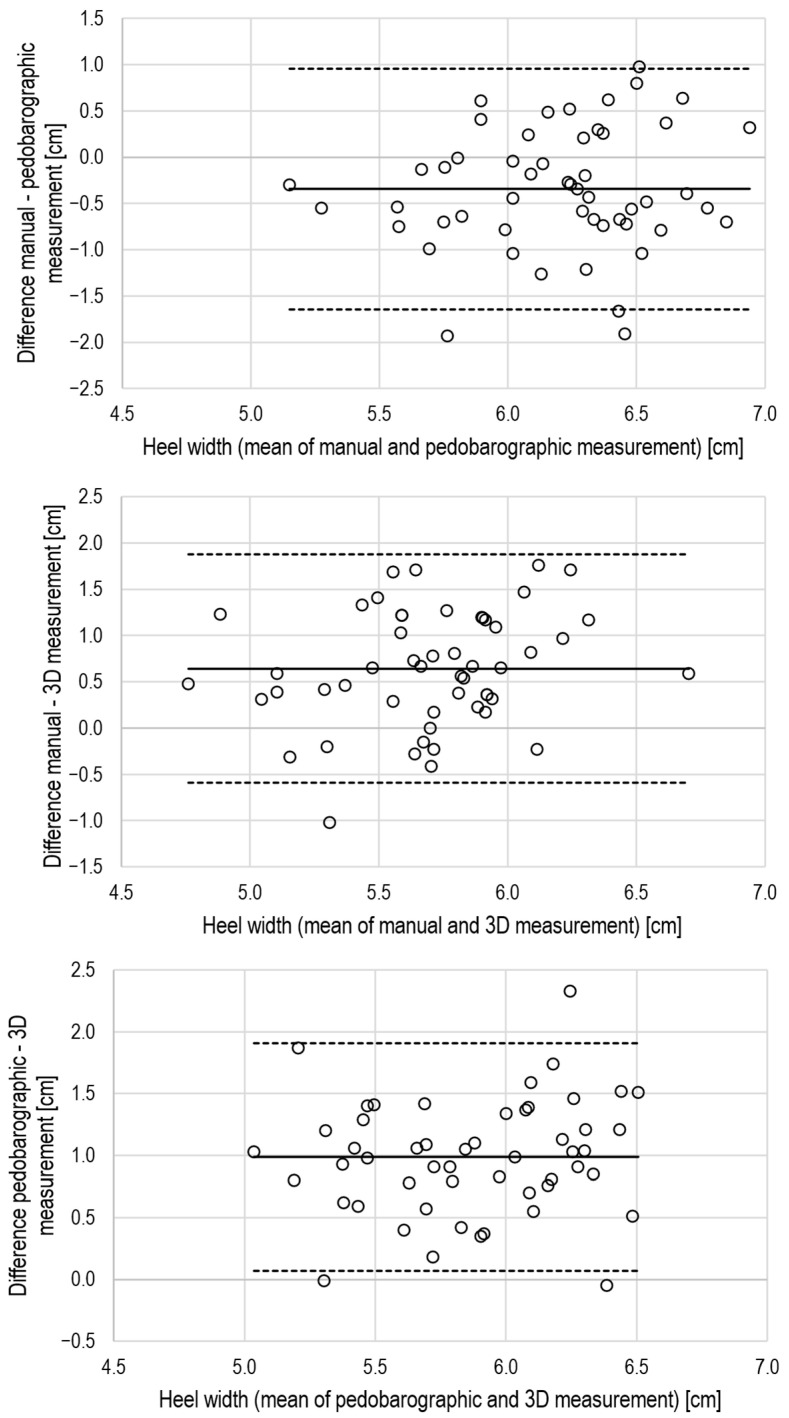
Bland–Altman plots for the evaluation of intermethod agreement for heel width, solid lines: mean difference, dashed lines: limits of agreement (mean ± 2 × standard deviation), upper panel: manual vs. pedobarography, middle panel: manual vs. 3D scanning, lower panel: pedobarography vs. 3D scanning.

The CCC values indicated excellent agreement (CCC ≥ 0.8) for foot length, moderate agreement (CCC > 0.2 and < 0.8) for forefoot width, and poor agreement for heel width (CCC < 0.2) when comparing manual with pedobarographic measurements. Further, there was a small but systematic bias for foot length, while heel width differed not only relevantly but also significantly between manual and pedobarographic measurements (Table 3). The Bland–Altman plots also support the view of limited agreement between manual and pedobarographic measurements (Figure 4, Figure 5 and Figure 6).

The CCC values indicated moderate agreement for foot length and forefoot width when comparing manual measurements with 3D scanning. The agreement of heel width measurements was poor (Table 3). There were also systematic and large biases for foot length and forefoot width, as well as a large but non-significant bias for heel width. The Bland–Altman analysis supports the view of a limited agreement between manual and 3D measurements by relevant biases and large limits of agreement (Figure 4, Figure 5 and Figure 6).

Based on the CCC values, a moderate agreement between pedobarography and 3D scanning can be concluded for foot length, forefoot width, plantar arch width, and arch index. All dimensions, except foot length and plantar arch width, suffered from systematic bias between pedobarography and 3D scanning (Table 3, Figure 4, Figure 5 and Figure 6).

## 4. Discussion

This study evaluated the relative and absolute reliability of manual examination, pedobarography, and handheld 3D scanning for assessing key foot dimensions under weight-bearing conditions and provides estimates of the agreement between these commonly used measurement techniques.

Good-to-excellent relative and absolute intra-rater reliability was found for the distance-related foot dimensions across all three methods, with the exception of absolute reliability of heel width assessed with pedobarography. Relative and absolute reliability of the angular parameters assessed via pedobarography and 3D scanning ranged from poor to excellent, depending on the specific parameter and measurement method. The forefoot angle showed poor relative and absolute test–retest reliability for pedobarographic measurements. Both the forefoot angle and the intermetatarsal angle demonstrated poor relative and absolute test–retest reliability when assessed using 3D scanning. Relative and absolute intra-rater reliability for the distance-related dimensions obtained by 3D scanning under full weight-bearing conditions in our study was comparable to manual examination as the clinical gold standard. This is consistent with findings from other studies [3,11,12,13,25] (Table 4). These investigations also reported excellent ICC values (>0.90) for smaller dimensions such as heel width. In contrast to the handheld 3D device applied in our study, measurements using stationary 3D systems may benefit from merged and processed data from multiple high-resolution lasers. Previous studies also examined footprint angles with 3D scans, but reliability data only exist for the hallux valgus angle, for which Chen et al. reported good intra-rater reliability [3]. Other footprint angles are typically assessed using pressure platforms with better spatial sensor resolution, but their relative intra-rater reliability varies widely, which is consistent with our findings (ICC: 0.33–0.78) [3,26].

Inter-method agreement between manual examination, pedobarography, and 3D scanning ranged from poor to good based on the CCC values. No foot dimension demonstrated interchangeable results across techniques when considering all three indices, the CCC, mean bias, and the limits of agreement. The largest discrepancies were observed for heel width and arch-related measures. Excellent agreement between 3D scanning and manual examination has been reported for foot length and forefoot width [27]. To the best of our knowledge, no previous studies have compared 3D scanning and pedobarography regarding foot dimensions or angular parameters. The wide range of agreement (from excellent to poor, based on CCC, mean bias, and Bland–Altman analyses) observed in the present study may be explained by differences in the impact of body weight on foot dimensions [28]. During dynamic pedobarography, participants’ foot dimensions were measured during walking. This likely generated higher ground reaction forces compared to static weight-bearing, resulting in increased tissue deformation and larger length and width values [28]. Differences in foot alignment between gait and quiet standing further resulted in smaller dynamic angles such as the hallux valgus angle [29]. Interestingly, footprint data from static 3D scans fell between the results of manual examination and dynamic pedobarography. This may indicate that tissue deformation or physical activity prior to testing influenced the measured differences [28]. Generally, it has to be considered that the interpretation of the applied CI-based cut-off values [20] is very strict. Further, the sample variance for most of the measured parameters is small, and thus, correlation analysis results in reduced coefficients per se.

The clinical relevance of the observed measurement differences must also be considered. Variability in linear measures such as foot length or forefoot width was small and is unlikely to influence clinical decision-making. In contrast, larger discrepancies in heel width and arch-related parameters may affect foot-type classification or the evaluation of deformities, particularly near diagnostic thresholds. Angular measures showed similar variability, which may influence clinical interpretation when assessing structural alignment. These findings underline that measurements obtained with different techniques should not be used interchangeably without standardisation.

However, Bland–Altman analyses also suggest that only foot length can be regarded as interchangeable, while agreement between manual examination, pedobarography, and 3D scanning appears poor for forefoot and heel width. Here, the limits of agreement exceeded 20% of the mean for forefoot width and more than 100% for heel width.

Practical considerations such as cost, accessibility, and time efficiency must also be taken into account when evaluating handheld 3D scanning as a clinical tool. Manual examination is inexpensive and widely accessible but operator-dependent. Pedobarography requires specialised equipment, fixed installation, and trained personnel [13]. Handheld 3D scanners are more expensive than manual tools but remain substantially less costly and more flexible than stationary 3D systems [10,11]. Scanning can be completed within seconds and requires only moderate user training. Combined with software-assisted processing, handheld 3D scanning offers a compelling balance between precision and clinical applicability [11].

A limitation of this study is that inter-rater reliability was not assessed. Each measurement technique was carried out by a single trained examiner (one for 3D scanning and one for pedobarography), which ensured methodological consistency but did not enable comparisons between different raters [13,23]. Future research should therefore examine whether comparable results can be achieved across examiners to test external validity.

Although the sample was relatively small, its size is comparable to previous methodological studies using pedobarographic assessments, typically including 20–40 participants [6].

## 5. Conclusions

The study’s results indicate that handheld 3D scanning provided good-to-excellent relative and absolute intra-rater reliability for most distance-related foot dimensions and for the arch index, whereas angular parameters demonstrated poor to moderate reliability. Agreement between handheld 3D scanning and established methods such as manual examination or pedobarography was low, indicating that these approaches cannot be used interchangeably. Although handheld 3D scanners offer substantially greater flexibility, their diagnostic value currently lies mainly in capturing linear weight-bearing foot dimensions. Future work integrating computer-aided evaluation and automated processing may further improve the accuracy and clinical usability of 3D-based foot assessments.

## Figures and Tables

**Table 1:** Foot dimensions determined by manual examination, pedobarography, and 3D scanning.

	*Means ± Standard Deviations*		
	Manual	Pedobarography	3D Scanning
**Foot length [cm]**	27.9 ± 1.2	28.3 ± 1.5	27.0 ± 1.3
**Forefoot width [cm]**	10.6 ± 0.7	10.7 ± 0.5	10.0 ± 0.6
**Heel width [cm]**	6.0 ± 0.5	6.4 ± 0.4	5.4 ± 0.4
**Plantar arch width [cm]**	−	3.1 ± 0.8	2.9 ± 0.9
**Subarch angle [°]**	−	97.1 ± 8.2	102.9 ± 6.9
**Forefoot angle [°]**	−	111.6 ± 4.6	114.3 ± 3.6
**Intermetarsal angle [°]**	−	3.7 ± 0.5	5.8 ± 1.8
**Arch index**	−	0.2 ± 0.1	0.2 ± 0.1

**Table 2:** Relative (intraclass correlation coefficients [ICCs] with 95% confidence intervals [CI]) and absolute intra-rater reliability indices (bias, limits of agreement, typical error) for the assessment of footprint parameters by manual examination, pedobarography, and 3D scanning.

		*Bias*	*SD*	*p*	*Limits of Agreement*		*Typical Error*	*N*	*ICC (95% CI)*
Dimension	Technique				Lower	Upper			
**Foot length [cm]**	**Manual**	−0.115	0.306	0.009	−0.727	0.48	0.216	52	0.981 (0.958–0.992)
	**Pedobarography**	−0.032	0.284	0.419	−0.600	0.52	0.201	52	0.995 (0.989–0.998)
	**3D Scanning**	−0.033	0.470	0.601	−0.972	0.89	0.332	52	0.982 (0.959–0.992)
**Forefoot width [cm]**	**Manual**	−0.012	0.221	0.685	−0.455	0.42	0.156	52	0.983 (0.962–0.992)
	**Pedobarography**	0.103	0.479	0.126	−0.855	1.04	0.339	52	0.903 (0.787–0.956)
	**3D Scanning**	−0.093	0.291	0.020	−0.675	0.48	0.206	52	0.954 (0.890–0.980)
**Heel width [cm]**	**Manual**	−0.019	0.257	0.591	−0.533	0.48	0.182	52	0.951 (0.890–0.978)
	**Pedobarography**	−0.020	0.477	0.764	−0.975	0.91	0.338	52	0.810 (0.573–0.915)
	**3D Scanning**	−0.011	0.312	0.795	−0.634	0.60	0.220	52	0.872 (0.713–0.943)
**Plantar arch width [cm]**	**Pedobarography**	0.012	0.482	0.858	−0.952	0.96	0.341	52	0.966 (0.924–0.985)
	**3D Scanning**	−0.048	0.528	0.497	−1.104	0.99	0.373	52	0.951 (0.892–0.978)
**Subarch angle [°]**	**Pedobarography**	0.638	6.075	0.462	−11.513	12.55	4.296	52	0.890 (0.748–0.952)
	**3D Scanning**	1.375	6.098	0.097	−10.821	13.33	4.312	52	0.820 (0.604–0.919)
**Forefoot angle [°]**	**Pedobarography**	1.064	6.100	0.105	−11.136	13.02	4.313	52	0.450 (−0.205–0.751)
	**3D Scanning**	−0.500	6.039	0.190	−12.578	11.34	4.270	52	0.573 (0.063–0.807)
**Intermeta-tarsal angle [°]**	**Pedobarography**	0.009	0.281	0.817	−0.553	0.56	0.199	52	0.960 (0.912–0.982)
	**3D Scanning**	0.930	1.724	0.000	−2.518	4.31	1.219	52	0.624 (0.157–0.832)
**Arch index**	**Pedobarography**	0.005	0.021	0.064	−0.036	0.05	0.015	52	0.955 (0.920–0.974)
	**3D Scanning**	0.002	0.042	0.722	−0.082	0.08	0.030	52	0.791 (0.547–0.904)

*Bias:* mean difference between repeated measurements, *p*: two-sided significance, *SD*: standard deviation.

**Table 3:** Lin’s Concordance Correlation Coefficients (CCC) with 95% confidence intervals (CI) and mean bias for evaluating inter-method agreement between manual examination, pedobarography, and 3D scanning.

	*CCC (95% CI)*		
Dimension	Manual vs. Pedobarography	Manual vs. 3D Scanning	3D Scanning vs. Pedobarography
Foot length [cm]	0.858 (0.779–0.910)	0.662 (0.539–0.757)	0.661 (0.550–0.749)
Forefoot width [cm]	0.614 (0.420–0.754)	0.477 (0.301–0.621)	0.445 (0.293–0.574)
Heel width [cm]	0.127 (−0.095–0.338)	0.091 (−0.053–0.230)	0.137 (0.05–0.220)
Plantar arch width [cm]	−	−	0.760 (0.620–0.853)
Subarch angle [°]	−	−	0.271 (0.060–0.459)
Forefoot angle [°]	−	−	0.098 (−0.124–0.310)
Intermetatarsal angle [°]	−	−	0.037 (−0.023–0.097)
Arch index	−	−	0.660 (0.481–0.785)
	***Bias ± Standard Deviation (2-Sided Significance)***		
**Dimension**	**Manual—pedobarography**	**Manual—3D Scanning**	**Pedobarography—3D Scanning**
Foot length [cm]	−0.3 ± 0.7 (*p* = 0.004)	−1.1 ± 0.6 (*p* < 0.001)	1.4 ± 0.5 (*p* = 0.641)
Forefoot width [cm]	−0.1 ± 0.6 (*p* = 0.104)	0.5 ± 0.6 (*p* < 0.001)	0.7 ± 0.4 (*p* < 0.001)
Heel width [cm]	−0.3 ± 0.7 (*p* < 0.001)	−0.6 ± 0.6 (*p* = 0.336)	1.0 ± 0.5 (*p* < 0.001)
Plantar arch width [cm]	−	−	0.1 ± 0.7 (*p* = 0.144)
Subarch angle [°]	−	−	−5.2 ± 8.4 (*p* < 0.001)
Forefoot angle [°]	−	−	−2.4 ± 5.3 (*p* = 0.001)
Intermetatarsal angle [°]	−	−	−2.0 ± 1.8 (*p* < 0.001)
Arch index	−	−	−0.01 ± 0.04 (*p* = 0.012)

**Table 4:** Intraclass correlation coefficient (ICC) values for footprint parameters, angles, and indices in comparable reliability studies using manual and 3D scanning systems.

	Lee et al. [13]		Ballester et al. [11]		Rogati et al. [12]		Chen et al. [3]
	Caliper	3D ^1^ *	Caliper	3D ^1^ *	Caliper	3D ^2^ *	3D ^3^ *
Foot length	0.98	0.98	0.99	0.99	0.99	0.99	
Forefoot width	0.74	0.95	0.99	0.99	0.99	0.99	
Heel width	0.87	0.94					
Arch index						0.67	0.91

* 3D scanning systems: ^1^ INFOOT Inc., Tokyo, Japan ^2^ Kinect 3D foot scanner, Redmond, US ^3^ FAST, Enford International Co., Taichung Cit, Taiwan.

## Data Availability

The data from this study are available from the corresponding author upon reasonable request.
